# Cerato-Platanin Induces Resistance in *Arabidopsis* Leaves through Stomatal Perception, Overexpression of Salicylic Acid- and Ethylene-Signalling Genes and Camalexin Biosynthesis

**DOI:** 10.1371/journal.pone.0100959

**Published:** 2014-06-26

**Authors:** Ivan Baccelli, Lara Lombardi, Simone Luti, Rodolfo Bernardi, Piero Picciarelli, Aniello Scala, Luigia Pazzagli

**Affiliations:** 1 Department of Agri-food Production and Environmental Sciences, University of Florence, Florence, Italy; 2 Department of Biology, University of Pisa, Pisa, Italy; 3 Department of Biomedical, Experimental and Clinical Sciences, University of Florence, Florence, Italy; 4 Department of Agriculture, Food and Environment, University of Pisa, Pisa, Italy; University of Nebraska-Lincoln, United States of America

## Abstract

Microbe-associated molecular patterns (MAMPs) lead to the activation of the first line of plant defence. Few fungal molecules are universally qualified as MAMPs, and proteins belonging to the cerato-platanin protein (CPP) family seem to possess these features. Cerato-platanin (CP) is the name-giving protein of the CPP family and is produced by *Ceratocystis platani*, the causal agent of the canker stain disease of plane trees (*Platanus* spp.). On plane tree leaves, the biological activity of CP has been widely studied. Once applied on the leaf surface, CP acts as an elicitor of defence responses. The molecular mechanism by which CP elicits leaves is still unknown, and the protective effect of CP against virulent pathogens has not been clearly demonstrated. In the present study, we tried to address these questions in the model plant *Arabidopsis thaliana*. Our results suggest that stomata rapidly sense CP since they responded to the treatment with ROS signalling and stomatal closure, and that CP triggers salicylic acid (SA)- and ethylene (ET)-signalling pathways, but not the jasmonic acid (JA)-signalling pathway, as revealed by the expression pattern of 20 marker genes. Among these, *EDS1, PAD4, NPR1, GRX480, WRKY70*, *ACS6, ERF1a*/*b, COI1*, *MYC2*, *PDF1.2a* and the pathogenesis-related (PR) genes 1–5. CP rapidly induced MAPK phosphorylation and induced the biosynthesis of camalexin within 12 hours following treatment. The induction of localised resistance was shown by a reduced susceptibility of the leaves to the infection with *Botrytis cinerea* and *Pseudomonas syringae* pv. *tomato*. These results contribute to elucidate the key steps of the signalling process underlying the resistance induction in plants by CP and point out the central role played by the stomata in this process.

## Introduction

Microbe- or pathogen-associated molecular patterns (MAMPs/PAMPs) are microbe-derived molecules with a key role in the plant immune system: their perception by pattern recognition receptors (PRRs) leads to the activation of PAMP-triggered immunity (PTI), which is the first line of defence against pathogens [1].

In fungi, relatively few molecules are universally considered as MAMPs, such as chitin, with its variants like chitosan, ethylene-inducing xylanase, β-glucans, necrosis- and ethylene-inducing peptide 1 (Nep1)-like proteins and ergosterol [2]–[4]. Some of these molecules are not only produced by fungi, such as β-glucans and Nep1-lke proteins, which can be found in oomycetes and bacteria [5], [6]. Recently, proteins belonging to a new fungal protein family, the “cerato-platanin family”, have provided more and more experimental evidence of their MAMP activity.

Cerato-platanin proteins (CPPs) are produced by plant pathogenic and non-pathogenic fungi, both ascomycetes and basidiomycetes [7]. Concerning the primary role of these proteins, recent results suggest that they are mono-domain expansin-like proteins localised in the cell wall and involved in the hyphal growth and development [8]–[11]. Moreover, a role in the fungus-plant interaction has also been reported [12]. When CPPs are applied on host and non-host plants, they induce defence-related responses and resistance against pathogens [13]–[16].

Cerato-platanin (CP) is the name-giving protein of this family and is produced by *Ceratocystis platani*, the causal agent of the canker stain disease of plane trees (*Platanus* spp.) [17]. After surface treatment of plane tree leaves, CP induces defence-related responses and programmed cell death [18], [19]. In addition, CP has been reported to induce the production of phenolic compounds in various plants [17], [20], [21].

The mechanism by which CP elicits host and non-host plants after foliar treatment is still poorly understood, because the protein is unable to penetrate the leaf cuticle and a receptor has not been identified yet [22]. The data present in the literature about other CPPs do not help to unravel the question because these proteins are usually injected into the tissues by infiltration. Also the signalling pathways activated in the plants by CP or CPPs are unknown, although an involvement of the plant hormone salicylic acid has been recently reported in tobacco after treatment with BcSpl1 from *Botrytis cinerea* [13].

In the present study we aimed to clarify the questions concerning the resistance-inducing mechanism triggered by CP in the model plant *Arabidopsis*. In particular, we investigated the role played by the stomata, the activation of the signalling pathways and the biosynthesis of the phytoalexin camalexin following foliar treatment with CP. In addition, the ability of CP to induce localised resistance against *Botrytis cinerea* and *Pseudomonas syringae* pv. *tomato* was analysed.

## Results

### The production of H_2_O_2_ after treatment with CP spreads from stomata to the neighbouring cells

As the oxidative burst is one of the first responses occurring in plants after MAMP recognition [2], we assessed the ability of CP to elicit *Arabidopsis* leaves by analysing the production of hydrogen peroxide. H_2_O_2_ was first analysed in situ by treating the lower (abaxial) leaf surface according to a previous work performed on *P. acerifolia* [19]. H_2_O_2_ was visualised by the fluorescent probe 2,7-dichlorofluorescin diacetate (H_2_DCF-DA), a compound that easily enters the cells [19]. Fluorescence was already visible after 15 min of treatment (Fig. S1), and some stomata were more fluorescent than the background. After 1 h the fluorescence was already very intense, differently from what occurred when we treated the upper (adaxial) surface of the leaf, which developed a weak fluorescence at this treatment time (data not shown). Leaves remained fluorescent for 24 h. The production of H_2_O_2_ in situ was also analysed by treating leaves with serial dilutions of CP, for 15 and 60 min, and CP was able to induce H_2_O_2_ production even at a lower concentration (75 µM) (data not shown).

**Figure 1:**
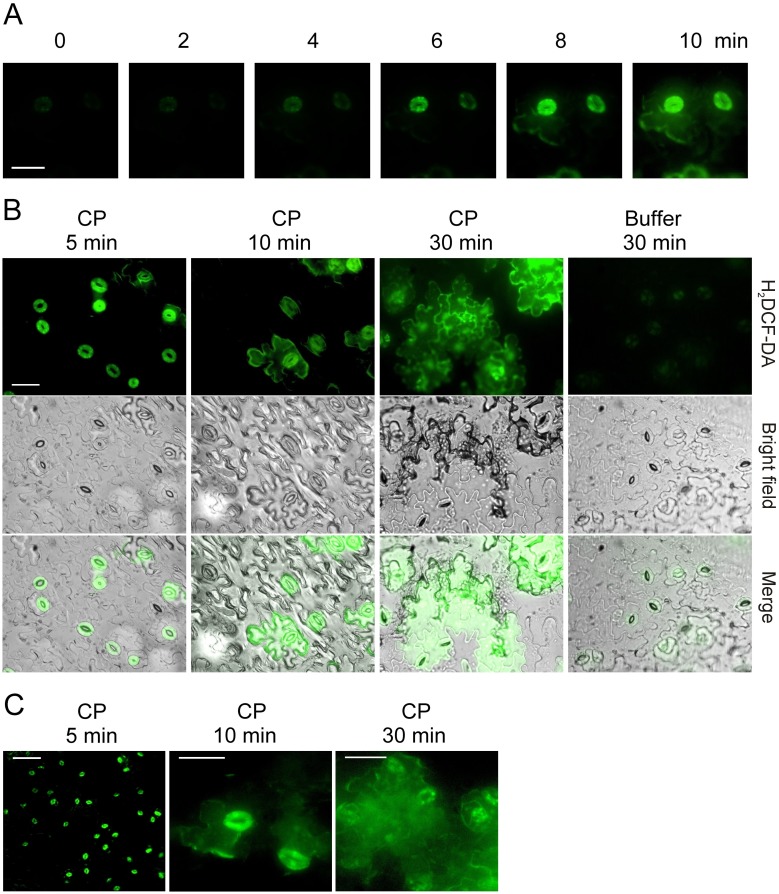
H_2_O_2_ production at the level of stomata. Epidermal peels from *Arabidopsis* leaves were first loaded with the fluorescent probe H_2_DCF-DA and then treated either on the cuticle side (A and B) or on the underside (C) with 150 µM CP to analyse the origin of the ROS signalling. (A) Single guard cells photographed at 2-minute intervals after treatment with CP (same peel). Bar = 30 µm. (B) Photographs of representative regions of three different peels treated with CP for 5, 10 or 30 min respectively, or 30 min with MES buffer (control). Fluorescence microscopy (H_2_DCF-DA), light microscopy (bright field) and merged pictures (merge) are shown in order to facilitate the localisation of stomata. The bar is 40 µm and applies to all photographs. (C) Peels treated on the underside (the side devoid of cuticle) with CP. Bars = 100 µm (5 min) and 30 µm (10 and 30 min).

Subsequently, we investigated stomata as a signalling source of H_2_O_2_ and we used epidermal peels to analyse the evolution of the H_2_O_2_ production on the epidermis. Epidermal peels were first loaded with the fluorescent probe, washed, and then treated on the cuticle side with CP. This procedure allowed the probe to be present in all the cells before the treatment, so that the fluorescence evolution only depended on the H_2_O_2_ production over time. H_2_O_2_ production by single stomata over time and on the same peel is shown in Fig. 1A. Guard cells turned out to be the first cells on the epidermis to become fluorescent, starting to synthesise H_2_O_2_ after 4–6 min of treatment. Before that time, the fluorescence was only due to the oxidizing environment of the chloroplast [23]. After 8–10 min the epidermal cells surrounding the stomata also became fluorescent. Because of the impossibility of analysing a single peel under the microscope for a longer period, we decided to repeat the experiment with different epidermal peels. Peels were treated for 5, 10 and 30 min. As shown in Fig. 1B, once again the first fluorescence signals occurred in the stomata, while after 30 min the fluorescence had spread to most epidermal cells. In the same experiment, closed stomata did not produce H_2_O_2_ (Fig. S2A). In addition, no fluorescence was observed in a 2-h period when we treated epidermal peels obtained from plants with stomata still closed (Fig. S2B).

In order to exclude the possibility that the guard cells responded faster to CP because devoid of cuticle, we treated epidermal peels on the underside (where the cuticle is not present). Once again, the H_2_O_2_ production started in the stomata and only subsequently spread to the neighbouring epidermal cells (Fig. 1C).

### CP induces stomatal closure

**Figure 2:**
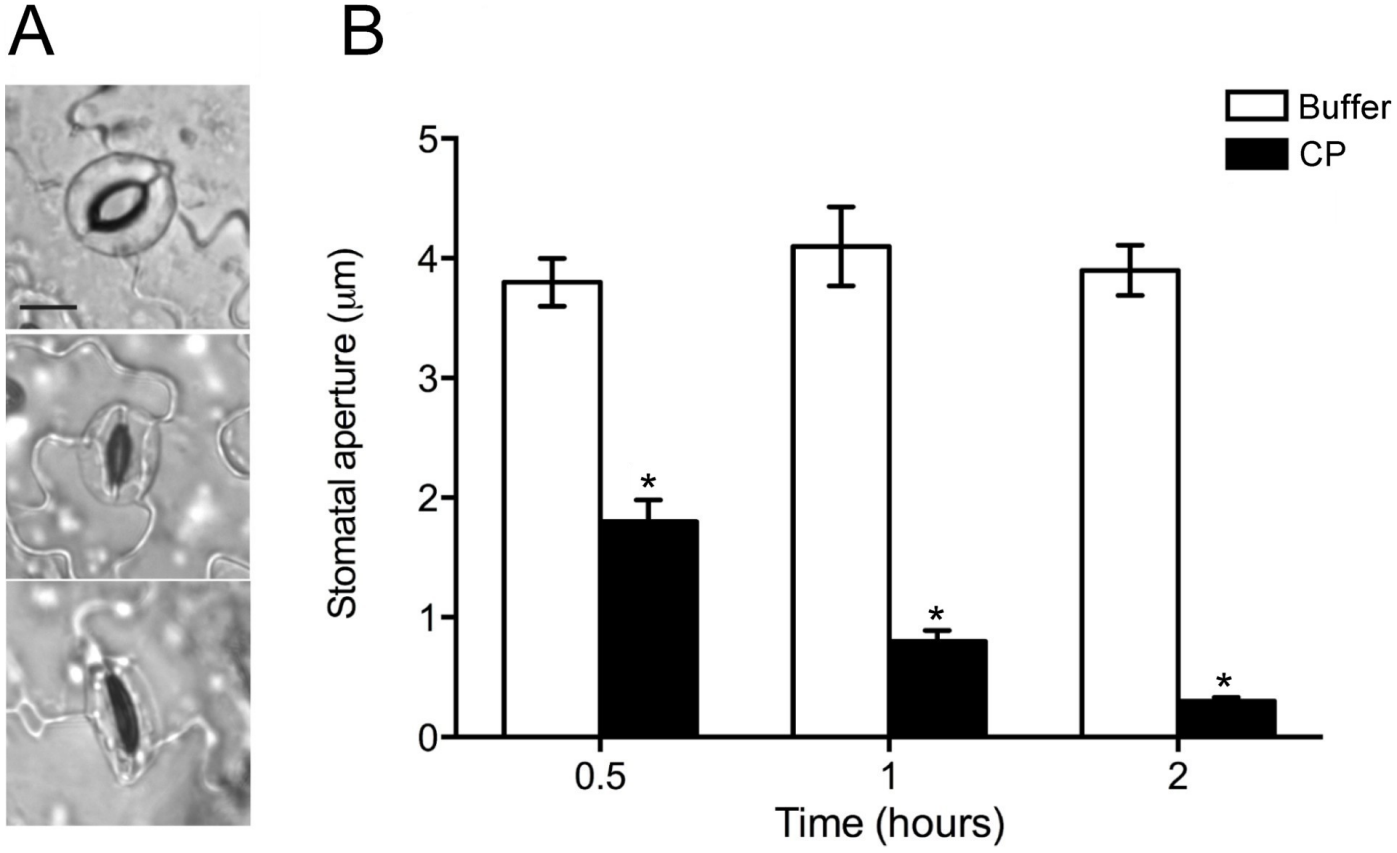
Stomatal closure induced by CP. (A) Open and closed stomata in *Arabidopsis* epidermal peels. Bar = 10 µm. (B) Measurement of stomatal aperture after 30 min, 1 and 2 h of treatment with 150 µM CP or MES buffer (control). Results are the mean of 100 measurements ± SEM. Statistical analysis was performed by unpaired *t*-test (treated vs. control). Asterisks indicate statistically significant difference at *P*<0.05. The experiment was repeated with similar results.

According to previous studies performed with elicitors different from CPPs [24], [25], we used epidermal peels, which allow better microscopic recording, to investigate the ability of CP to influence the stomatal movement (Fig. 2A). As shown in Fig. 2B, CP induced a progressive reduction in the average width of the stomatal aperture. The reduction was already significant after 30 min, and after 2 h the stomata were almost completely closed. The treatment with MES buffer, which was used as a control, did not influence the stomatal movement.

### Phosphorylation of MAP kinases

The activation of the MAPK cascade following treatment with CP was studied in *Arabidopsis* by analysing the phosphorylation of MAPK3 and MAPK6, according to the recent evidence that indicates a key role of the kinase induction in *Arabidopsis* defence responses [26]–[28]. For example, MPK3 and MPK6 play roles in camalexin induction, the major phytoalexin in *Arabidopsis*, and posses a critical role in priming *Arabidopsis* plants for full induction of defence responses during induced resistance [29], [30].

**Figure 3:**
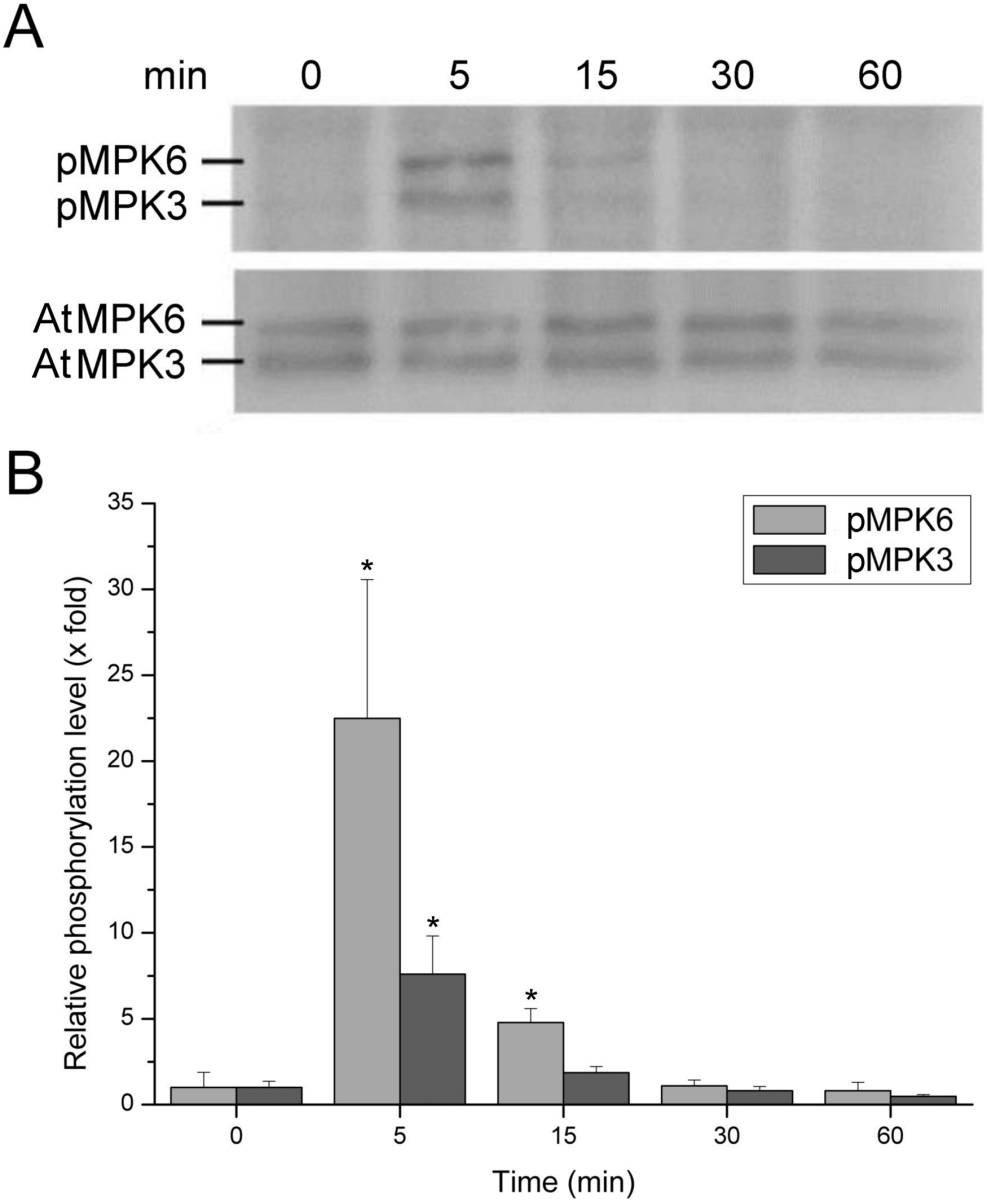
Phosphorylation of *Arabidopsis* MPK6 and MPK3 after treatment with 150 µM CP. (A) Analysis carried out with human phospho-p44/42 antibodies (pERK1/2) on protein extracts obtained after 0, 5, 15, 30 and 60 min of treatment. pMPK6 and pMPK3 indicate phosphorylation. (B) Image analysis showing the phosphorylation level normalized to the respective protein amount. Error bars indicate SD of three biological replicates. Statistical analysis was performed by unpaired *t*-test by comparing the phosphorylation level to the appropriate background level (0 h). Asterisks indicate statistically significant difference at *P*≤0.05.

The analysis was carried out with specific phospho-p44/42 (pERK1/2) antibodies on protein extracts obtained after 0, 5, 15, 30 and 60 min of treatment with CP. As shown by immuno-blot analysis, both MPK3 and MPK6 turned out to be rapidly phosphorylated (Fig. 3A). After only 5 min of treatment MPK6 was phosphorylated up to 20-fold the control value (Fig. 3B). As expected, MAPKs were not phosphorylated when leaves were treated with sterile distilled water (data not shown).

### CP up-regulates SA- and ET-signalling genes, but not JA-signalling genes

As a first approach, we used a primer library purchased from Sigma-Aldrich containing gene-specific primers for pathogen-inducible genes. The study was performed after 6 h of treatment, which was the time in which the greatest number of genes was expected to be modulated (preliminary data not shown). By this analysis, we identified 56 up-regulated genes out of 67 genes analysed (Table S1). It is worth mentioning genes involved at different levels with the oxidative stress, such as glutathione *S*-transferases (*GSTF3, GSTF7*), peroxidase 50, late embryogenesis abundant-like protein 5 (*LEA5*), blue copper protein (*BCB*) and respiratory burst oxidase homologue D (*RBOHD*) [31]–[34]; or genes codifying for various receptor kinases, suchs as cysteine-rich receptor-like protein kinases (*CRK5*, *CRK7*, *CRK10*, *CRK36*), lectin-receptor kinase, receptor-like protein kinase 1 (*RLK1*), wall-associated receptor kinase 1 (*WAK1*) and somatic embryogenesis receptor kinase 4 (*SERK4*) [35]. Many other up-regulated genes could instead be related to defence-signalling pathways, mainly SA and ET. As the signalling pathways activated by CPPs in *Arabidopsis* are unknown, we focused our attention on the expression of SA-, ET- and JA-dependent genes.

**Figure 4:**
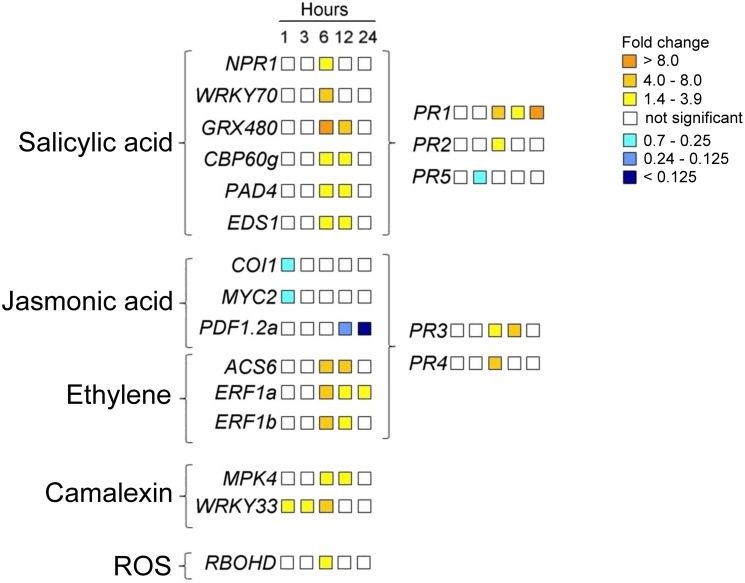
Expression analysis of genes related to defence-signalling pathways, camalexin synthesis and ROS production. Significant variations (*P*≤0.05) in the level of expression are reported in the figure with a colour scale (up-regulated genes = yellow; down-regulated genes = blue). *Arabidopsis* leaves were treated on the lower surface with 150 µM CP (treated sample) or sterile distilled water (calibrator sample) for 1, 3, 6, 12 or 24 h. *Actin-2* was used as the endogenous reference gene. Relative gene expression values (2^−ΔΔCt^ or fold change values) from three biological replicates and two technical replicates are shown. Statistical analysis was performed by unpaired *t*-test (treated sample vs. calibrator sample per each time point). The qPCR results and the locus ID of each gene can be seen in Table S1.

In order to perform a detailed study, we designed 13 more primer pairs to include, in the analysis, some of the most studied genes that we found in the literature. The study was performed at various treatment times (1, 3, 6, 12 and 24 h) on a total of 20 genes (7 were from the Sigma-Aldrich primer library). Among these, we analysed the expression of *MAPK4* and the transcription factor *WRKY33*, which act upstream of the synthesis of camalexin [36], and *RBOHD* (respiratory burst oxidase homologue D), which is implicated in the production of reactive oxygen species (ROS) [33]. The expression pattern of the analysed genes is shown in Fig. 4. CP induced the expression of SA- and ET-dependent genes starting from 6 h after the treatment, and this was also the treatment time in which most genes were found modulated following application of CP. This did not occur for JA-dependent genes, which were instead down-regulated by CP. *COI1* (coronatine insensitive 1) and *MYC2* (jasmonate insensitive 1, *JIN1*), two key regulators of JA signalling [37], were down-regulated after only 1 h of treatment, while *PDF1.2a* (plant defensin) was strongly down-regulated after 12 and 24 h.

The expression of pathogenesis-related (PR) genes was analysed taking into account their differential expression in SA- and JA-/ET-signalling pathways [38]. As shown in Fig. 4, PR1, PR2 (β-1, 3-glucanase), PR3 (basic chitinase) and PR4 (hevein-like protein) turned out to be all up-regulated by CP, starting from 6 h after the treatment. However, their up-regulated status was differently maintained. In particular, PR1 was up-regulated until 24 h. PR5 (thaumatin-like) was instead down-regulated.

*WRKY33* turned out to be the earliest up-regulated gene (1 h) by CP and its status was maintained until 6 h; *MAPK4* was up-regulated after 6 h and its status was maintained until 12 h. Finally, *RBOHD* was up-regulated after 6 h of treatment.

### CP induces the biosynthesis of camalexin

**Figure 5:**
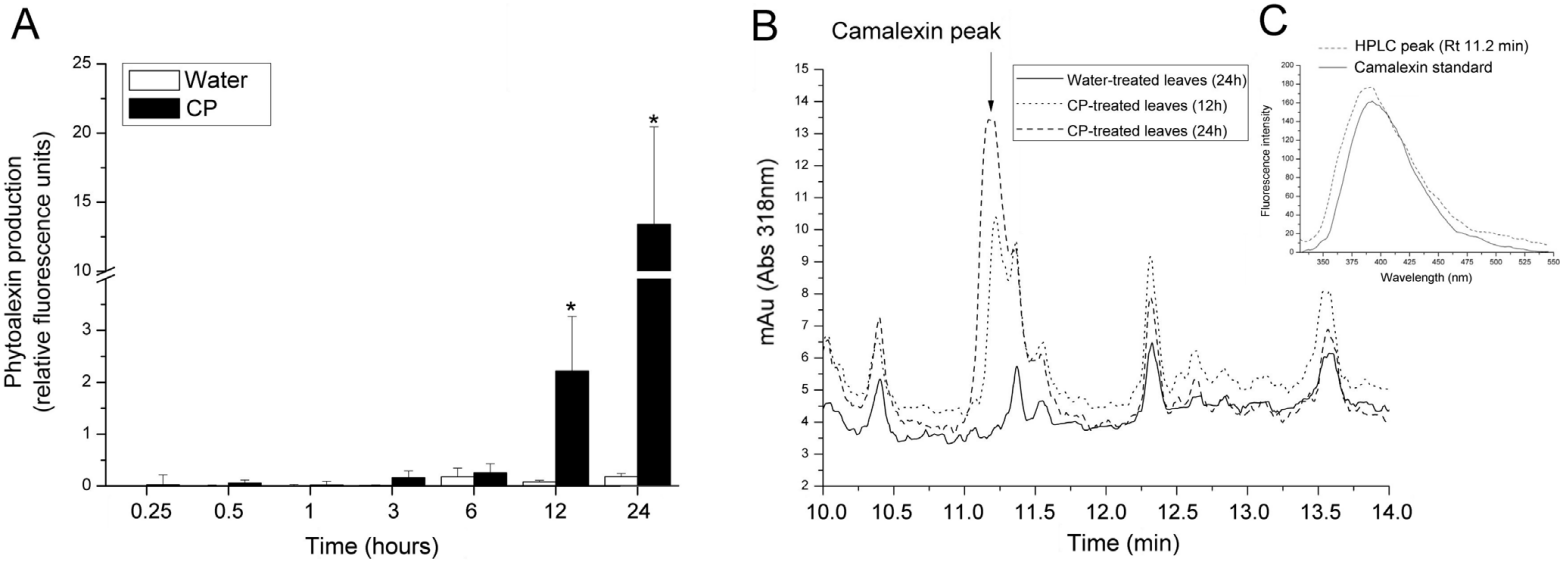
Phytoalexin production by *Arabidopsis* leaves treated with CP. (A) Leaves treated on the lower surface with 150 µM CP or sterile distilled water (control). The drops were collected after 15 and 30 min, 1, 3, 6, 9, 12 or 24 h of treatment and the phytoalexin release measured by fluorescence analysis (λex = 320 nm, λem = 386 nm). The fluorescence value was normalized to the number of droplets analysed and expressed as relative fluorescence units. Error bars indicate SD of three biological replicates. Statistical analysis was performed by unpaired *t*-test (treated vs. control). Asterisks indicate statistically significant difference at *P*<0.05. (B) Reverse Phase-High Performance Liquid Chromatography (RP-HPLC) of camalexin extracted from the leaves treated for 12 and 24 h. The retention time (Rt) of the peak indicated by the arrow is the same as the camalexin standard, as experimentally determined. (C) Fluorescence emission spectrum of the peaks eluted at 11.2 min compared to a camalexin standard.

Phytoalexins are phenolic compounds whose production after treatment with CP has been reported in different plants as a release of fluorescence substances [17], [20], [21]. Nevertheless, the biochemical nature of these substances has never been investigated in detail. In the present study, the synthesis of phytoalexins was first determined in *Arabidopsis* leaves by measuring the fluorescence of the droplets recovered from the leaves. As shown in Fig. 5A, the fluorescence in the droplets significantly increased starting from 12 h following treatment and became about six times higher after 24 h. In order to determine whether the fluorescence was due to camalexin biosynthesis, the emission peak of a camalexin standard was compared to the peak obtained from the recovered droplets. As shown in Fig. S3, the two spectra almost overlapped, thus suggesting that the fluorescence emitted after treatment with CP was due to camalexin production. To confirm this observation and to investigate the presence of camalexin in the tissues, treated leaves were extracted with methanol and analysed by reverse-phase chromatography (Fig. 5B). The analysis revealed the presence of a main peak (with a retention time of 11.2 min) that matches the retention time of a camalexin standard analysed with the same method (Fig. S4). To further confirm the result, the eluted peak at 11.2 min was recovered and its fluorescence emission compared to the standard (Fig. 5C). The overall results indicate that camalexin was produced by *Arabidopsis* leaves following treatment with CP.

As these results were obtained by treating the lower leaf surface, we compared the two leaf surfaces in their ability to release phytoalexins after treatment with CP. After 48 h of treatment, the fluorescence emitted by the droplets recovered from the lower surface was about double than that emitted by the droplets recovered from the upper surface (Fig. S5).

### CP protects *Arabidopsis* leaves from *Botrytis cinerea* and *Pseudomonas syringae* pv. *tomato*

In order to assess the effectiveness of CP in protecting the plant from pathogens, *Arabidopsis* leaves were treated on the lower surface with 10-µl drops of CP, sterile distilled water (negative control) or 0.1% chitosan (positive control). After 24 h of treatment, the drops were recovered and the leaves were infected on the same foliar surface with virulent pathogens.

**Figure 6:**
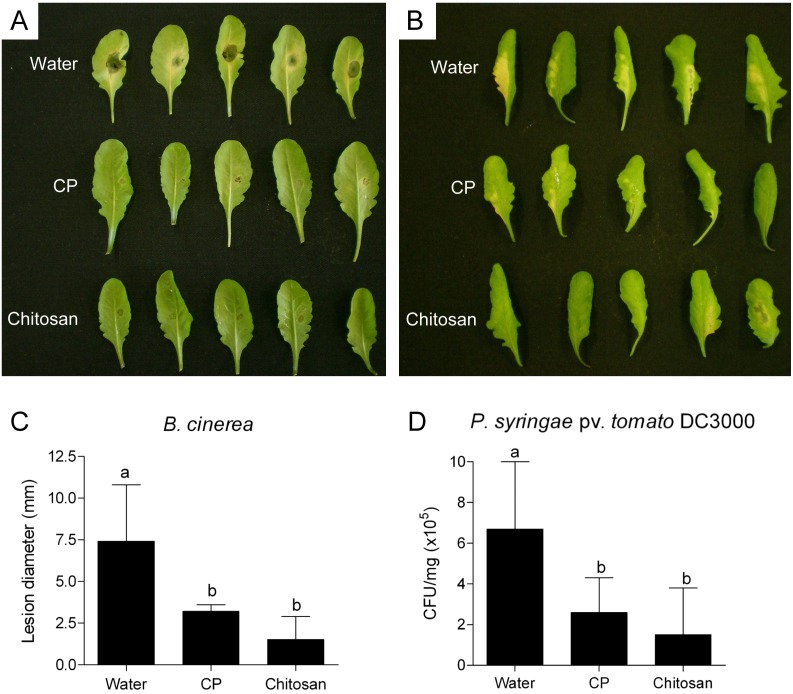
Resistance induction assays against *Botrytis cinerea* and *Pseudomonas syringae* pv. *tomato* (*Pst*). *Arabidopsis* detached leaves were treated on the lower surface with 150 µM CP, sterile distilled water (control) or 0.1% chitosan for 24 h before pathogen inoculation. (A) *B. cinerea* strain PM10 was inoculated by placing a single 10-µl drop of a suspension 2×10^5^ conidia ml^−1^ in 1% Sabouraud Maltose Broth on a side of the middle vein. Infected leaves were incubated for 3 days at 22°C before taking photos and measuring the lesion size. (B) *Pst* strain DC3000 was inoculated by placing a single 10-µl drop of a suspension 10^8^ colony-forming units (CFU) ml^−1^ (OD_600_ = 0.2) in sterile distilled water containing 0.02% Silwet L-77 on a side of the middle vein. Infected leaves were incubated for 3 days at 28°C before taking photos and determining bacterial titer. (C) Measurement of the lesion diameter (mm) ± SD after 3 days of incubation with *B. cinerea* (n = 8). (D) Counting of CFU mg^−1^±SD after 3 days of incubation with *Pst* DC3000 (n = 8). Statistically significant differences among treatments are indicated at *P*≤0.05.

*B. cinerea* strain PM10 was inoculated by placing a single 10-µl drop of a suspension 2×10^5^ conidia ml^−1^ on a side of the middle vein. After 3 days of incubation at 22°C, CP-treated leaves showed a consistent reduction of the disease symptoms (Fig. 6A, C). The lesion size in CP-treated leaves was statistically similar to what occurred after the treatment with chitosan. The lesions remained basically limited to the point of application of the conidial suspension, although after the treatment with chitosan some leaves showed a complete absence of lesions.

*P. syringae* pv. *tomato* (*Pst*) strain DC3000 was similarly inoculated by placing on the leaf surface a single 10-µl drop of a suspension 10^8^ colony-forming units (CFU) ml^−1^. After 3 days of incubation at 28°C, the leaves were photographed and the bacterial titer was determined. As shown in Fig. 6B, the treatments with CP and chitosan reduced both symptom development and bacterial growth (Fig. 6D) compared to water-treated leaves. Chitosan appeared more effective than CP in limiting the growth of *Pst* DC3000, but the difference compared to CP was again not significant.

No direct inhibiting effect of CP against the germination of *B. cinerea* conidia or the growth of *Pst* DC3000 was found when the pathogens were grown in vitro in the presence of 150 µM CP (data not shown).

## Discussion

ROS are central players in the complex signalling network of cells and it is well known that MAMPs cause an oxidative burst upon their recognition by the plant [2]. An initial burst of ROS production can trigger a cascade of cell-to-cell communication events that propagate the signal over long distances like a wave [39]. In the present study, we analysed the production of H_2_O_2_ after treatment with CP at the level of stomata. We treated *Arabidopsis* leaves on the external epidermal surface for two reasons: the external application mimics the first natural contact between a MAMP and the plant tissue, and above all it can be representative of a possible use of CP in plant protection.

Hypothesizing a role for stomata in the perception mechanism of CP, we assumed that H_2_O_2_ had to be produced first by the stomata. Accordingly, we designed the experiment to monitor the H_2_O_2_ evolution on the epidermis and we started the analysis almost instantaneously (2 min). To our knowledge, there are no other data in the literature that show the initial production of H_2_O_2_ at the level of stomata and the subsequent spreading on the epidermis after surface treatment with microbial-derived elicitors. Among the CPPs, the H_2_O_2_ production in *Arabidopsis* leaves has been reported for MgSM1 and BcSpl1 [16], [40]. However, MgSM1 had been assessed 24 h after the ectopic expression in transgenic plants, whereas BcSpl1 had been assessed 4 h after the infiltration.

Our results suggest that CP elicited defence responses by entering through the stomata, and perhaps its perception occurred at the level of the inner surface of the guard cells. This action model was suggested by some clear observations: the H_2_O_2_ production after the treatment with CP was initiated by guard cells (4–6 min) and only subsequently spread from the stomata to the neighbouring epidermal cells (not *vice versa*); this occurred even when the peels were treated on the underside, which is devoid of cuticle; the treatment of the lower leaf surface, which normally presents a higher stomatal density [41], [42], resulted in a higher production of H_2_O_2_ and phytoalexins; the treatment performed with closed stomata did not result in H_2_O_2_ production. Accordingly, we can deduce that (a) stomata have a key role in the *Arabidopsis* immunity triggered by CP; (b) the guard cells probably possess a receptor for this MAMP. This assumption is in accordance with the role in MAMP sensing attributed to guard cells by Melotto *et al.* [25], who also hypothesized the presence in these cells of several receptors for multiple MAMPs, and with the actual presence in these cells of the flagellin receptor [43]. At the same time our results do not conflict with the presence of a receptor also in the plasma membrane of epidermal or mesophyll cells, as recently suggested by Frías *et al.* [44] for the CPP BcSpl1. However, at the level of epidermal cells our results strongly suggest that the stomata are the most responsive to CP. In fact, even when the peels were treated on the underside, i.e. the side devoid of cuticle, the H_2_O_2_ production showed the same evolution, namely from guard cells to neighbouring cells, and thus the presence of cuticle cannot be considered the only determinant of the obtained results.

Stomata represent a natural entry site for potentially harmful microbes, and the stomatal closure in response to MAMPs is considered as an innate immune response active at the pre-invasive level [25], [45]. CP induced a progressive reduction of the stomatal aperture similarly to flagellin-22 (flg22) and lipopolysaccharides [25]. The generation of H_2_O_2_ is crucial to initiate this process and MAMPs rely on RBOHD as the primary NADPH oxidase for ROS production [45], [46]. As found by qPCR, *RBOHD* was effectively up-regulated by CP but the timing (6 h) could not explain the early oxidative burst observed. Therefore, we can assume that the rapid generation of H_2_O_2_ was caused by this enzyme in the form already present in the cells.

The activation of MAPK cascades is an early response to MAMP signals [2]. CP rapidly induced the phosphorylation of MAPK3 and MAPK6. This result was expected from previous results obtained in *P. acerifolia* leaves [19] and confirms that the activation of the kinase cascade is one of the first events in plant defence signalling.

In order to establish the signalling events triggered by CP, we performed an extensive gene expression analysis never performed before for CPPs. We analysed 17 genes related to SA-, JA- and ET-signalling pathways, which are basically new in relation to the CPPs.

We found that the SA-dependent and the ET-dependent genes, whose roles and reciprocal interactions in the signalling pathways have been reported in the literature [47]–[49], showed the same activation time (6 h). However, the intensity and the duration of the overexpression varied depending on the gene. On the contrary, the down-regulation of the JA-dependent genes occurred almost instantaneously (1 h), as revealed by *COI1*, the JA receptor [50], and *MYC2*, a transcription factor involved in the transcriptional regulation of some JA-responsive genes [51]. The lack of activation of the JA signalling could also be confirmed by the subsequent down-regulation of the plant defensin *PDF1.2a* [52]. Confirming the well-known antagonism between SA and JA signalling [53], our result leads us to predict a role for SA and ET, rather than JA, in CP-induced defence signalling in *Arabidopsis*. In tobacco, Frías *et al.* [13] have recently reported a role for SA after treatment with the CPP BcSpl1. However, this is the first time that a role for ET signalling has been reported.

*MAPK4* and *WRKY33* were analysed for their involvement in the biosynthesis of camalexin, the major phytoalexin in *Arabidopsis* [36], [54]. Phytoalexins are secondary metabolites that show biological activity against a variety of pathogens and are considered molecular markers of disease resistance [54]. Interestingly, the resistance induced by elicitors in *Arabidopsis* to *B. cinerea* is independent of SA, ET, or JA signalling, whereas the biosynthesis of camalexin and other secondary metabolites seems to have a prominent role [55], [56]. CP up-regulated *MAPK4* and *WRKY33*, and induced the biosynthesis of fluorescent phenolic compounds identified as camalexin. The camalexin level was increased both inside the leaves treated with CP and in the droplets recovered from the leaf surface, starting from 12 h after treatment.

The present study was concluded by demonstrating the ability of CP to induce resistance against pathogens. To date the effectiveness of CP against pathogens had only been demonstrated against *C. platani* [57]. In that report, plane tree leaves treated with CP hampered *C. platani* germination and growth when inoculated with conidia. However *C. platani* is not a foliar pathogen. In this study we demonstrated that CP effectively enhances resistance against virulent foliar pathogens on the non-host plant *A. thaliana*. CP induced disease resistance to the necrotrophic fungus *B. cinerea* and the hemibiotrophic bacterium *P. syringae* pv. *tomato*.

In conclusion, with the results obtained in the present study we can assume that CP induces resistance in *Arabidopsis* leaves through stomata, which very rapidly sense CP and respond to the treatment with ROS signalling and stomatal closure. The signalling pathways triggered by CP certainly involve both SA and ET, as revealed by the expression pattern of marker genes, while JA does not seem to be involved. Biotrophic and hemibiotrophic pathogens are generally sensitive to defence responses that are regulated by SA, whereas pathogens with a necrotrophic lifestyle are commonly deterred by defences that are controlled by JA and ET [37], [47]. Accordingly, *P. syringae* pv. *tomato* was likely hampered by the activation of the SA-signalling pathway, whereas the activation of the ET-signalling pathway combined with the camalexin production were probably crucial for the outcome of the protection against *B. cinerea*. The results shown in the present study not only contribute to elucidate the key steps of the signalling process underlying the resistance induction in plants by CP but also point out the crucial role played by the stomata in the early signalling of microbial-derived molecules on the epidermis.

## Materials and Methods

### Production and purification of cerato-platanin

Cerato-platanin (CP) was produced by heterologous expression in the yeast *Pichia pastoris* [58], purified by C-18 Reverse Phase-High Performance Liquid Chromatography (RP-HPLC), quantified by the bicinchoninic acid (BCA) method (Pierce, Rockford, IL) and checked for purity by SDS-PAGE and mass spectrometry [59].

### Plant material and growth conditions

*Arabidopsis thaliana* plants, ecotype Col-0, were grown in soil in a growth chamber at 22°C with a 12-h photoperiod under light intensity of 100 µE m^−2 ^s^−1^ for 4–5 weeks. The phosphorylation assay for MAP kinases was performed on 12-day-old seedlings as described below. Unless differently specified, the experiments were performed on intact detached leaves.

### Detection of hydrogen peroxide

The production of H_2_O_2_ in intact leaves after the treatment with CP was visualized in situ with the probe H_2_DCF-DA 10 µM (Sigma-Aldrich, St Louis, MO, USA) according to Lombardi *et al.* [19]. Leaves from 4-week-old plants were detached and treated on the leaf surface with 10-µl drops (12–15 drops per leaf) of 150 µM CP and incubated in a moist chamber for 5, 15, 30 min, 1, 3, 6, 12, 24 h. At the end of the incubation period the leaves were incubated for 30 min with the probe, washed twice with buffer to remove the excess of fluorophore, and mounted on slides. Controls were prepared by treating leaves with both sterile distilled water and the protein bovine serum albumin (BSA) 150 µM. Photographs were taken with a QICAM digital CCD camera (QImaging, CA, USA) attached to a Nikon Eclipse T*i*-S Microscope, equipped with filter block FITC (λex 488, λem 502–540 nm).

In order to find the minimum active concentration of CP, leaves were treated with 1∶2 serial dilutions of CP (starting from 300 µM) and the production of H_2_O_2_ in situ was analysed after 15 and 60 min.

In order to analyse the H_2_O_2_ production on the epidermis around the stomata, epidermal peels were used. *Arabidopsis* plants were kept for at least 3 h under the light to ensure that most stomata were open before beginning the experiments, then epidermis was peeled off and placed on glass slides with the cuticle side in contact with MES buffer. Peels were pre-incubated for 2 h in MES buffer (25 mM MES-KOH pH 6.15, 10 mM KCl) under the light, soaked for 20 min in 10 µM H_2_DCF-DA diluted in MES buffer, washed three times in MES buffer, and then incubated with 150 µM CP for 5, 10 or 30 min. MES buffer was used as a negative control. The experiment was repeated by treating peels with closed stomata. As a further analysis, epidermal peels were treated with CP on the side devoid of cuticle.

In order to analyse H_2_O_2_ production at the level of single guard cells, an epidermal peel was placed on a glass slide with the cuticle side in contact with CP and was visualized in continuum under the microscope for 10 min; single stomata were photographed at 2-minute intervals.

### Stomatal closure assay

*Arabidopsis* plants were kept for at least 3 h under the light to ensure that most stomata were open before beginning the experiments. Epidermal peels were prepared from three different fully expanded leaves as described in the previous paragraph. In those conditions, peels have about 70% open stomata [25]. The tissues were placed on glass slides with the cuticle side in contact with MES buffer or with 150 µM CP dissolved in MES buffer. MES buffer was used as a negative control. At various time points (30, 60 and 120 min) pictures were taken of random regions and the width of the stomatal aperture was measured by using Image-Pro Plus 6.0 (Media Cybernetics, Bethesda, MD, USA).

### MAPK phosphorylation assay

*Arabidopsis* seeds were sterilized by treating them for 3 min in 70% ethanol, 3 min in 20% bleach with 0.1% Tween 20 and extensively washed with sterile distilled water. Subsequently, the seeds were incubated in the dark at 4°C for 1 day and then dispensed into a 24-well tissue culture plate (10 seeds per well) containing 1 ml of Murashige and Skoog Basal medium supplemented with 0.5% sucrose, pH 5.7. The plates were incubated at 22°C with a 12-h-light photoperiod (100 µE m^−2 ^s^−1^ intensity). After 8 days, the medium was replaced and the treatments were performed after four additional days. Seedlings were treated for 5, 15, 30 and 60 min by adding to the medium either CP to a final concentration of 150 µM or an equal volume of sterile distilled water as a control.

Protein extraction and immunoblot analysis were performed according to Galletti *et al.* [26]. In summary, proteins were extracted with a buffer containing 50 mM Tris-HCl (pH 7.5), 200 mM NaCl, 1 mM EDTA, 10 mM NaF, 2 mM sodium orthovanadate, 1 mM sodium molybdate, 10% (v/v) glycerol, 0.1% Tween 20, 1 mM phenylmethylsulfonyl fluoride, 1 mM dithiothreitol, and 1X protease inhibitor cocktail P9599 (Sigma-Aldrich). Fifteen µg of proteins per each sample were resolved on 12% polyacrylamide gel and transferred onto a PVDF membrane (BioRad, Hercules, CA, USA). Primary antibodies against MPK3 and MPK6 (Sigma-Aldrich), and against phospho-p44/42 MAP kinase (Cell Signaling Technologies), were used with horseradish peroxidase-conjugated anti-rabbit as a secondary antibody. Signal detection was performed by ECL western detection kit (GE Healthcare). Image analysis was performed with the software KODAK MI v.4.0.4 (KODAK Rochester, New York, USA).

### Gene expression analysis

A primer library containing 93 gene-specific primers for pathogen-inducible genes (Primer Library for Arabidopsis Pathogen-inducible Genes, Sigma-Aldrich) was employed for a large scale analysis that we performed by qPCR after 6 h of treatment. Thirteen more primer pairs (Table S2) were designed with Primer Express Software 3.0 (Applied Biosystems) for a detailed analysis of defence-signalling pathways that we performed at 1, 3, 6, 12 or 24 h. Leaves from 4-week-old plants were detached and treated on the lower surface with 10-µl drops of CP 150 µM (treated sample) or sterile distilled water (control sample) as previously described and incubated in a moist chamber for 1, 3, 6, 12 or 24 h. Three treated leaves and three control leaves were incubated per each time.

Total RNA was extracted by using GenElute Mammalian Total RNA Miniprep Kit (Sigma-Aldrich), treated with Amplification Grade DNase I (Sigma-Aldrich) and reverse-transcribed into cDNA (400 ng per sample) by using iScript cDNA synthesis kit (BioRad, Hercules, CA, USA).

Real-time reactions (20 µl) were carried out with 10 ng of cDNA, 250 nM primers, and 1×Fast SYBR Green Master Mix (Applied Biosystems, Foster City, CA, USA) following the manufacturer’s instructions. PCRs were run in a StepOne real-time PCR System (Applied Biosystems) by using the recommended thermal-cycling conditions (hold 95°C, 20 s; 40 cycles 95°C, 3 s; 60°C, 30 s). Before performing the relative expression calculation the performance of each amplification was checked and some primers were excluded from the study.

The relative gene expression values (2^−ΔΔCt^) were calculated by using *Actin-2* as the endogenous reference gene and water-treated leaves as the calibrator sample (control sample), following the calculation described by Livak and Schmittgen [60]. *Actin-2* was used as a reference gene after confirmation of its transcriptional stability in our experimental conditions (CP- vs. water-treated leaves) (data not shown). *Actin-2* primers were included in the primer library. Statistical analysis (treated vs. control) was performed by unpaired *t*-test by using GraphPad InStat v. 3.05 (GraphPad Software, San Diego, CA, USA).

### Phytoalexin synthesis assay and camalexin detection

Drops were collected from the lower leaf surface of CP- or water-treated leaves after 15, 30, 60 min, 3, 6, 9, 12 and 24 h of incubation and the phytoalexin measurement was performed by fluorescence analysis (λex = 320 nm, λem = 386 nm, slit 5) by using a Perkin Elmer spectrofluorimeter 650-10 S (Perkin Elmer, Wellesley, MA). In order to confirm the release of camalexin from the leaves, the fluorescence spectrum (λex = 320 nm, λem = 330–550 nm, slit 5) of the droplets collected after 24 h of incubation was compared to the spectrum obtained by using a pure camalexin standard (kindly provided by Dr. E. Glawischnig, University of Technology, Munich, Germany). The experiment was also repeated to compare the two leaf surfaces in their ability to release phytoalexins after 48 h of treatment.

Camalexin was extracted with methanol from CP-treated leaves (12 and 24 h) according to Beets and Dubery [61].

Ten µl of a standard stock solution of camalexin (15 µg ml^−1^ in methanol) and 40 µl of extracted sample were analysed with RP-HPLC using a C18 column, 3 µm, 15×4.6 cm (Supelco, Bellefonte, Pennsylvania, USA). Elution gradient was performed at a flow rate of 0.8 ml min^−1^ with the following solvent system: 10 mM trifluoroacetic acid (TFA) in acetonitrile (solvent A); 10 mM TFA in water (solvent B). The gradient used was 10% A for 2 min, from 10% to 98% A for 13 min, holding at 98% A for 10 min, from 98% to 100% A for 5 min. Detection was based on UV absorbance at 318 nm.

### Resistance induction assays

*Arabidopsis* leaves were detached from 5-week-old plants and treated on the lower surface with 10-µl drops of 150 µM CP, sterile distilled water (control) or 0.1% chitosan (about 20 µM in 10 mM acetic acid; molecular weight 50–60 kDa, deacetylation degree 85%). The leaves were incubated for 24 h in Petri dishes containing 0.8% agar, after that the drops were recovered and the leaves were infected on the same foliar surface with pathogens.

*Botrytis cinerea* strain PM10 [62] was grown on potato dextrose agar (PDA, Difco, Detroit, MI) at 22°C for 8 days before collecting conidia. Inoculations were performed by placing a single 10-µl drop per leaf of a suspension 2×10^5^ conidia ml^−1^ in 1% Sabouraud Maltose Broth (SMB) (maltose 8 g l^−1^, peptone 2 g l^−1^) on a side of the middle vein. Leaves were incubated at 22°C for 3 days before measuring the lesion diameter and taking photographs. In order to test for a direct inhibiting effect of CP against the germination of *B. cinerea* conidia, two drops of conidial suspension were placed on a glass slide and one of these was added with CP, whereas the other one was added with sterile distilled water for an equal volume. Germination and growth were monitored.

*Pseudomonas syringae* pv. *tomato* (*Pst*) strain DC3000 was cultured at 28°C, 300 rpm, in Luria-Bertani (LB; tryptone 10 g l^−1^, yeast extract 5 g l^−1^, NaCl 5 g L^−1^) medium supplemented with 50 mg l^−1^ rifampicin (Sigma-Aldrich) until an OD_600_ of 0.8 was reached (24–26 h). Bacteria were collected by centrifugation and resuspended in sterile water containing the surfactant Silwet L-77 0.02% (Momentive, Columbus, OH, USA) to the final concentration of 10^8^ colony-forming units (CFU) ml^−1^ (OD_600_ = 0.2). Inoculations were performed by placing a single 10-µl drop per leaf on a side of the middle vein. Leaves were incubated at 28°C for 3 days after that photographs were taken and the leaf bacterial titer was determined. The titer was determined by grounding the infected side of the leaf in 1 ml of 10 mM MgCl_2_ and by overspreading serial dilutions on LB agar (10 g l^−1^ agar) plates containing 50 mg l^−1^ rifampicin. CFU were counted after 2 days of incubation at 28°C. In order to test for a direct inhibiting effect of CP on the bacterial growth, *Pst* DC3000 was grown with 150 µM CP for 3 days.

Statistically significant differences among treatments were calculated by one-way ANOVA with Tukey-Kramer post test (*P*≤0.05) with GraphPad InStat v. 3.05 (GraphPad Software, San Diego, CA, USA).

## Supporting Information

Figure S1 **H_2_O_2_ production in** ***Arabidopsis*** **leaves treated with CP.** Leaves were treated on the lower (abaxial) surface with 10-µl drops of 150 µM CP or water (control) for 15 and 30 min, 1, 3, 6, 12 and 24 h. H_2_O_2_ was visualized in situ by the fluorescent probe 2,7-dichlorofluorescin diacetate (H_2_DCF-DA). The bar is 100 µm and applies to all photographs.
(TIF)

Figure S2 **The production of H_2_O_2_ only origins at the level of open stomata.** (A) Treatment of epidermal peels obtained from leaves with open stomata. Some closed stomata are indicated by the arrows. The peels were first loaded with the fluorescent probe H_2_DCF-DA and then treated on the cuticle side with 150 µM CP. (B) Treatment of epidermal peels obtained from leaves with closed stomata. Fluorescence microscopy (H_2_DCF-DA), light microscopy (bright field) and merged pictures (merge) are shown. The bar is 40 µm and applies to all photographs.
(TIF)

Figure S3 **Fluorescence spectrum of a pure camalexin standard and of sample of droplets collected from the foliar surface after 24 h of treatment.** Leaves were treated on the lower surface with 10-µl drops of 150 µM CP. The droplets were recovered and the fluorescence analysis was performed at λex = 320 nm.
(TIF)

Figure S4 **Retention time (Rt) of camalexin as revealed by RP-HPLC.** Standard stock solution of camalexin analysed by Reverse Phase-High Performance Liquid Chromatography (RP-HPLC). Rt = 11.2 min.
(TIF)

Figure S5 **Phytoalexin production by** ***Arabidopsis*** **leaves treated on the adaxial or abaxial surface with CP.** Leaves were treated with 10-µl drops of 150 µM CP or sterile distilled water (control) for 48 h. The phytoalexin release was measured by fluorescence analysis (λex = 320 nm, λem = 386 nm). The fluorescence value was normalized to the number of droplets analysed and was expressed as relative fluorescence units. Error bars indicate SD of three measurements. Statistical analysis was performed by unpaired *t*-test (treated vs. control). Asterisks indicate statistically significant difference at *P*<0.05.
(TIF)

Table S1 **Real-time RT-qPCR results.** Pathogen library (6 h) and time-course analysis (1, 3, 6, 12, 24 h) for selected genes.
(XLS)

Table S2 **Primers designed in the present study and used for the RT-qPCR analysis.**
(DOC)
