# Supplementary figures and images for: Cerato-Platanin Induces Resistance in Arabidopsis Leaves through Stomatal Perception, Overexpression of Salicylic Acid- and Ethylene-Signalling Genes and Camalexin Biosynthesis
Source: PLoS One. 2014 Jun 26;9(6):e100959. doi: 10.1371/journal.pone.0100959 (PMC4072723; doi:10.1371/journal.pone.0100959)

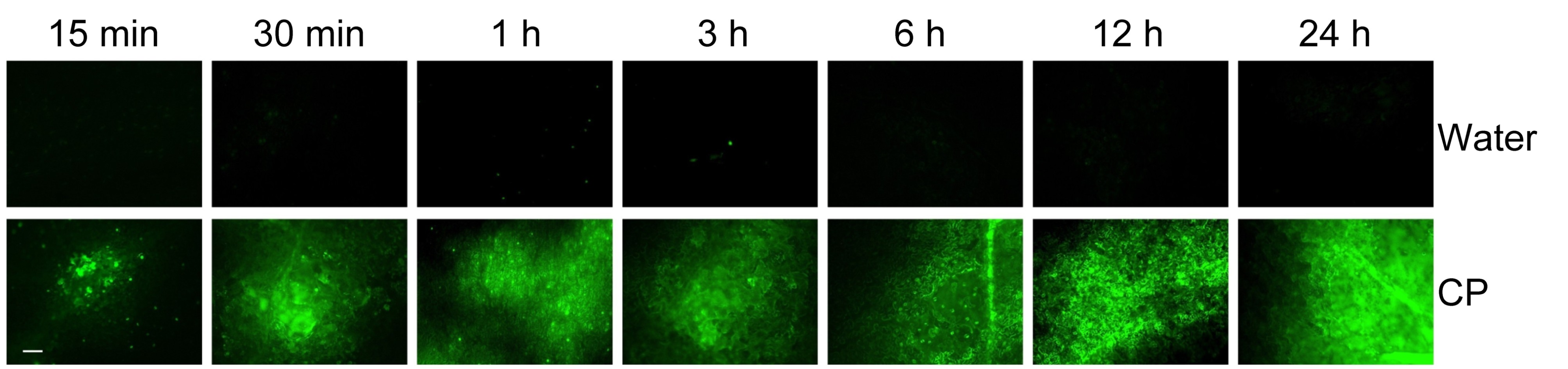

Supplement: Figure S1 — H2O2 production in Arabidopsis leaves treated with CP. Leaves were treated on the lower (abaxial) surface with 10-µl drops of 150 µM CP or water (control) for 15 and 30 min, 1, 3, 6, 12 and 24 h. H2O2 was visualized in situ by the fluorescent probe 2,7-dichlorofluorescin diacetate (H2DCF-DA). The bar is 100 µm and applies to all photographs. (TIF) [file pone.0100959.s001.tif]

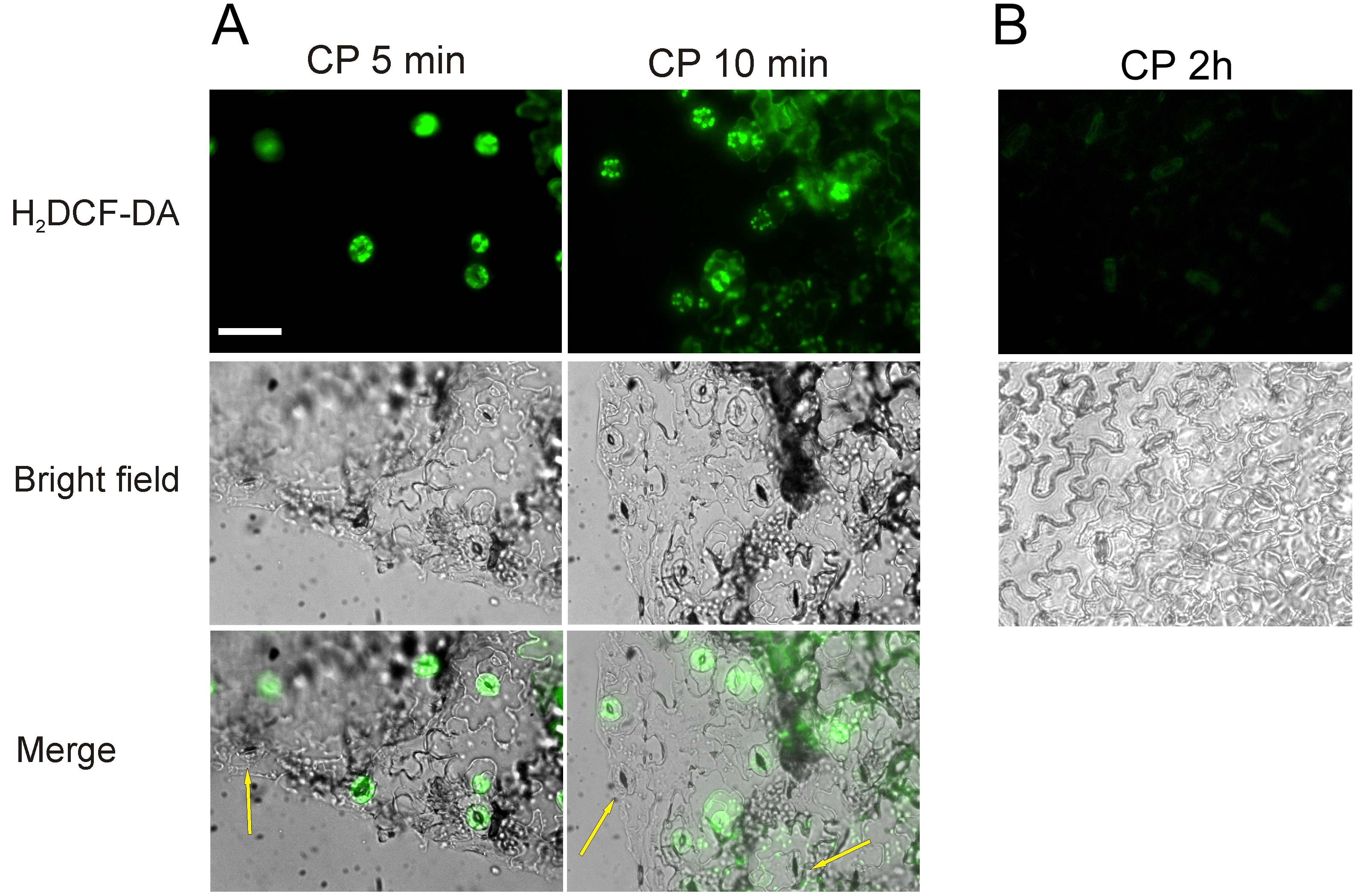

Supplement: Figure S2 — The production of H2O2 only origins at the level of open stomata. (A) Treatment of epidermal peels obtained from leaves with open stomata. Some closed stomata are indicated by the arrows. The peels were first loaded with the fluorescent probe H2DCF-DA and then treated on the cuticle side with 150 µM CP. (B) Treatment of epidermal peels obtained from leaves with closed stomata. Fluorescence microscopy (H2DCF-DA), light microscopy (bright field) and merged pictures (merge) are shown. The bar is 40 µm and applies to all photographs. (TIF) [file pone.0100959.s002.tif]

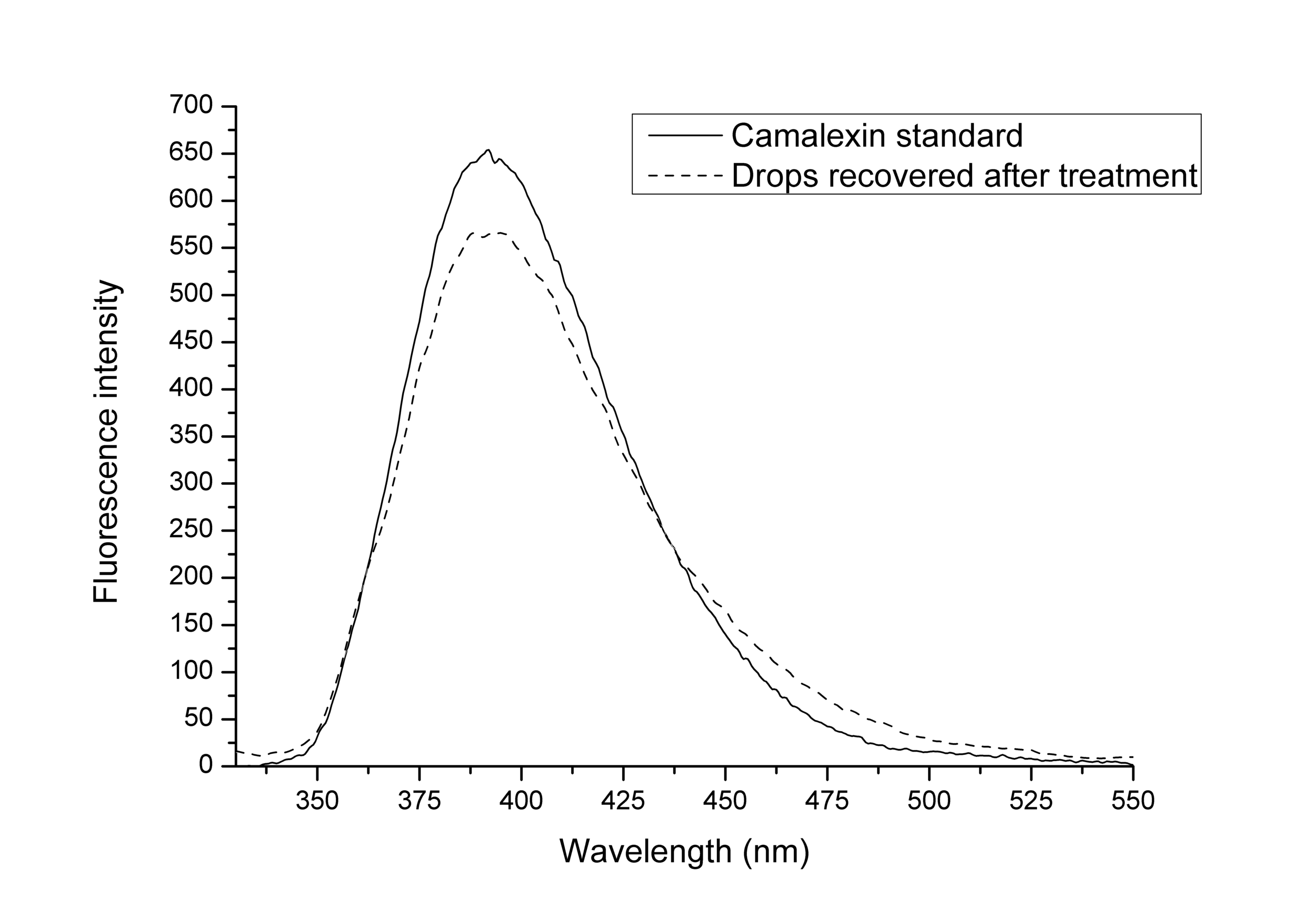

Supplement: Figure S3 — Fluorescence spectrum of a pure camalexin standard and of sample of droplets collected from the foliar surface after 24 h of treatment. Leaves were treated on the lower surface with 10-µl drops of 150 µM CP. The droplets were recovered and the fluorescence analysis was performed at λex = 320 nm. (TIF) [file pone.0100959.s003.tif]

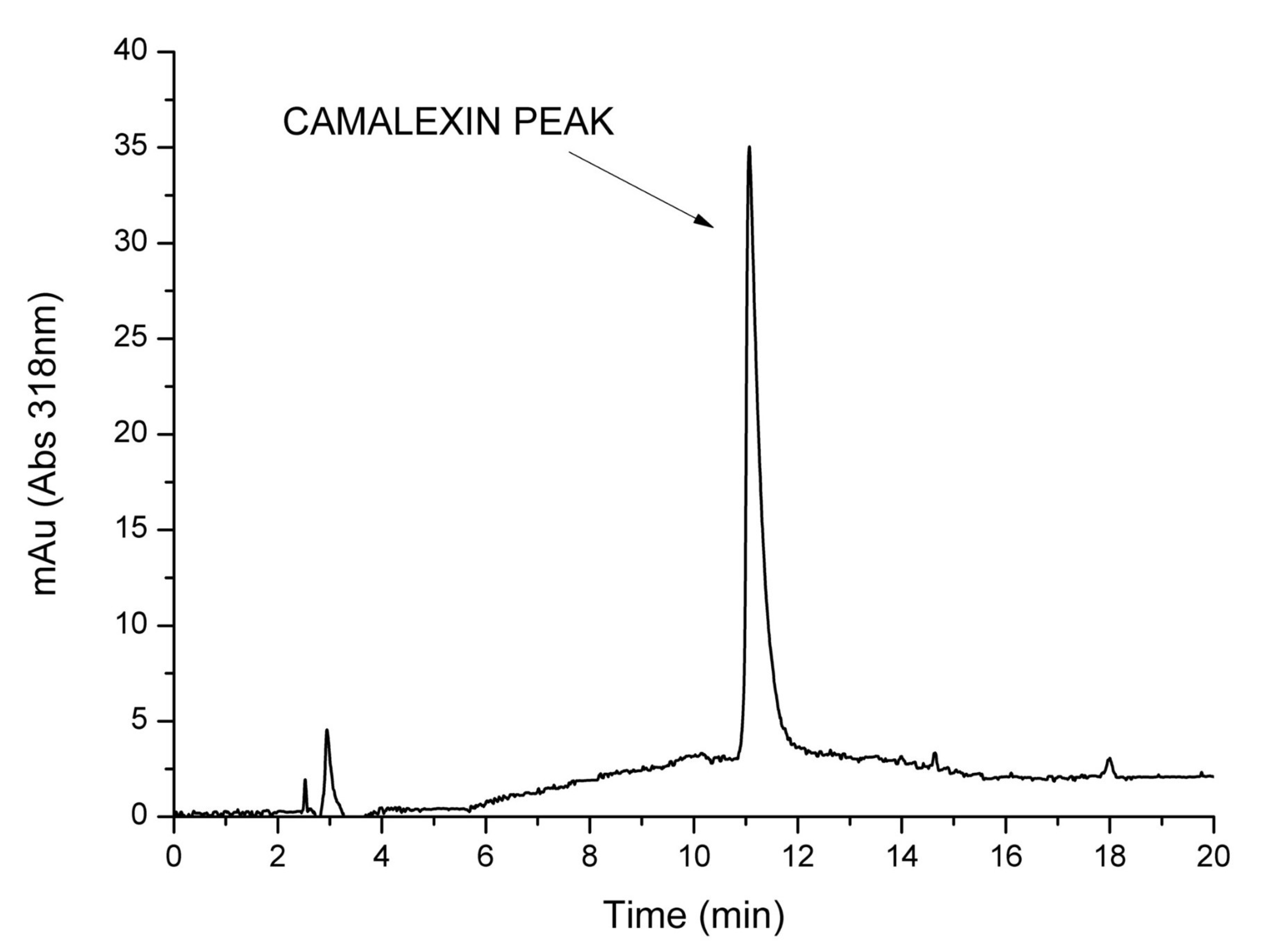

Supplement: Figure S4 — Retention time (Rt) of camalexin as revealed by RP-HPLC. Standard stock solution of camalexin analysed by Reverse Phase-High Performance Liquid Chromatography (RP-HPLC). Rt = 11.2 min. (TIF) [file pone.0100959.s004.tif]

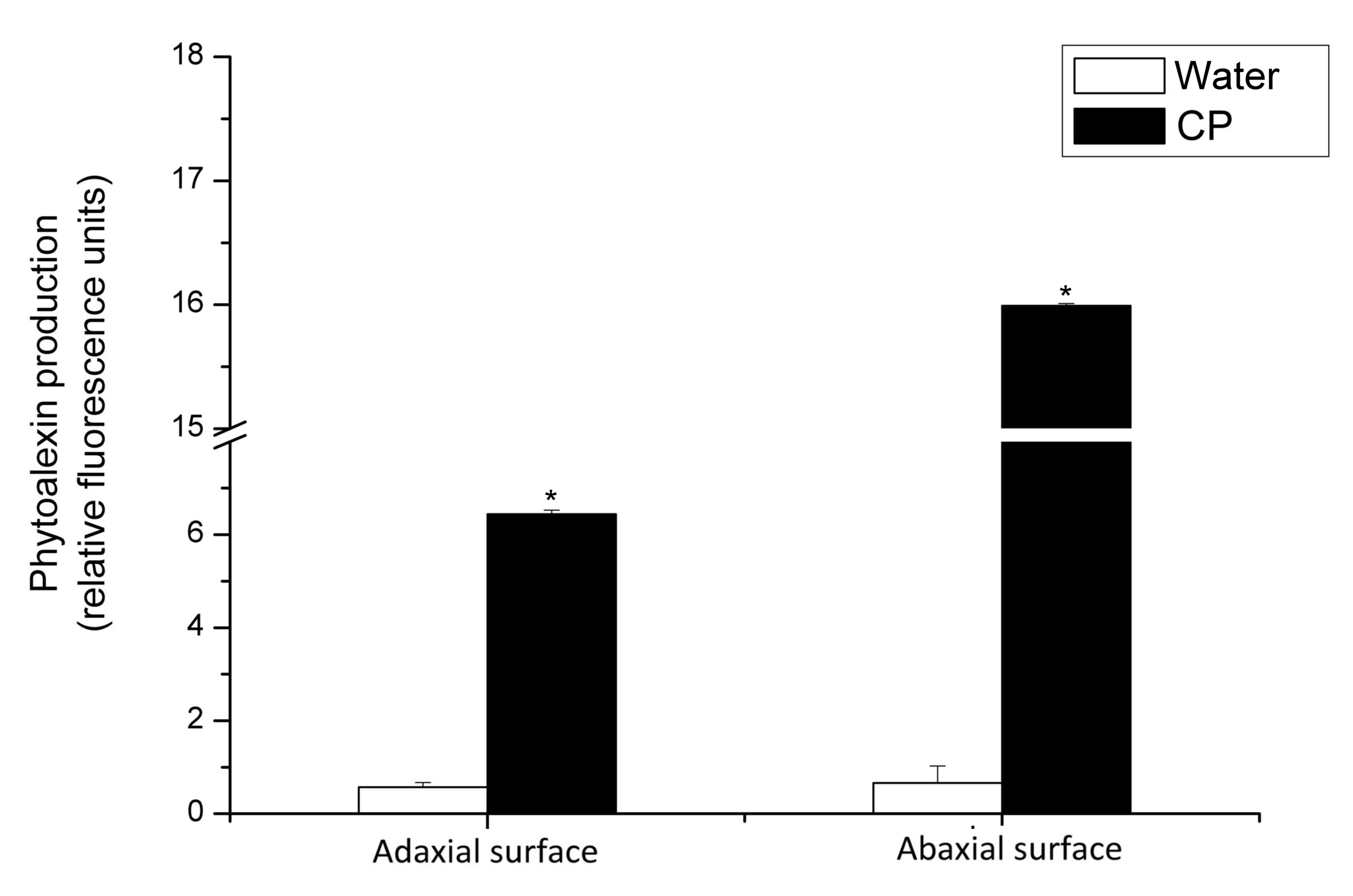

Supplement: Figure S5 — Phytoalexin production by Arabidopsis leaves treated on the adaxial or abaxial surface with CP. Leaves were treated with 10-µl drops of 150 µM CP or sterile distilled water (control) for 48 h. The phytoalexin release was measured by fluorescence analysis (λex = 320 nm, λem = 386 nm). The fluorescence value was normalized to the number of droplets analysed and was expressed as relative fluorescence units. Error bars indicate SD of three measurements. Statistical analysis was performed by unpaired t-test (treated vs. control). Asterisks indicate statistically significant difference at P<0.05. (TIF) [file pone.0100959.s005.tif]
