# Supplementary material for: Cerato-Platanin Induces Resistance in Arabidopsis Leaves through Stomatal Perception, Overexpression of Salicylic Acid- and Ethylene-Signalling Genes and Camalexin Biosynthesis
Source: PLoS One. 2014 Jun 26;9(6):e100959. doi: 10.1371/journal.pone.0100959 (PMC4072723; doi:10.1371/journal.pone.0100959)
Supplement: Table S2 — Primers designed in the present study and used for the RT-qPCR analysis. (DOC) [file pone.0100959.s007.doc]

**Table S1.** Primers designed in the present study and used for qPCR analysis

| **Gene** | **Locus** | **Primer sequence (5’-3’)** |
| --- | --- | --- |
| PR-1 | At2g14610 | Forward CTCGGAGCTACGCAGAACAAC  Reverse CGTAAGGCCCACCAGAGTGT |
| PR-2 | At3g57260 | Forward TCGACGTTCCCAGTTCAGATC  Reverse TGTCGGCCTCCGTTTGAC |
| PR-3 | At3g12500 | Forward ACGGAAGAGGACCAATGCAA  Reverse GGTCAGGGTTGTTGAGTAAGTCAA |
| PR-5 | At1g75040 | Forward TTGCTGTTATGGCCACAGACTT  Reverse TCCTTGACCGGCGAGAGTT |
| NPR1 | At1g64280 | Forward TGGAAGGTAGAACCGCACTCA  Reverse CTTGCATTGCTCCGGGATAT |
| GRX480 | At1g28480 | Forward TGCTTCTTGGACTTGGAGTGAA  Reverse TTCATCTTCCCTCTCCTCATCAA |
| EDS1 | At3g48090 | Forward TCAATGGCGTTTGAAGCTCTT  Reverse GGCTGACCATGATCTGGTGATT |
| MYC2 | At1g32640 | Forward CGGAATGGTTTTTCTTGGTTTC  Reverse ACCCGTTGCAAACGCTTTAC |
| COI1 | At2g39940 | Forward AAGATTGATTCCGAGACGAGAGA  Reverse GATCAGGCGTCGCAGTGTAG |
| PDF1.2a | At5g44420 | Forward GCTTCCATCATCACCCTTATCTTC  Reverse TTCTGTGCTTCCACCATTGC |
| ERF1b | At3g23240 | Forward GGTCCTCGGCGATTCTCAAT  Reverse AACGCCACAACCGGAGAAC |
| MPK4 | At4g01370 | Forward TAACGAAATGTCAACACCCATCTT  Reverse CGACATCGGAGCAAAATTCC |
| WRKY33 | At2g38470 | Forward CGGACAAAAACAGGTGAAAGG  Reverse CGTTGGACAATTAGGGAAAGTACA |
